# Serologic Evidence of Nipah Virus Infection in Bats, Vietnam

**DOI:** 10.3201/eid1803.111121

**Published:** 2012-03

**Authors:** Futoshi Hasebe, Nguyen Thi Thu Thuy, Shingo Inoue, Fuxun Yu, Yoshihiro Kaku, Shumpei Watanabe, Hiroomi Akashi, Dang Tuan Dat, Le Thi Quynh Mai, Kouichi Morita

**Affiliations:** Nagasaki University, Nagasaki, Japan (F. Hasebe, S. Inoue, F. Yu, K. Morita);; National Institute of Hygiene and Epidemiology, Hanoi, Vietnam (N.T.T. Thuy, L.T.Q. Mai);; National Institute of Infectious Diseases, Tokyo, Japan (Y. Kaku); University of Tokyo, Tokyo (S. Watanabe, H. Akashi);; Tay Nguyen Institute of Hygiene and Epidemiology, Dak Lak, Vietnam (D.T. Dat)

**Keywords:** Nipah virus, Nipah-like virus, henipavirus, bats, Vietnam, Rousettus leschenaulti, Cynopterus sphinx, ELISA, neutralization test, viruses

**To the Editor:** Bats are potential reservoir for highly pathogenic viruses, such as Nipah virus (NiV) and Hendra virus, which can cross species barriers (*1*). However, only limited surveillance has been conducted to assess risk for infection by these deadly emerging viruses. We conducted a study in Vietnam from 2007 to 2008 to assess the prevalence of these pathogens in bats.

Different species of live bats were obtained from hunters or were captured in caves, pepper fields, and residential areas by using mist nets or harp traps (Technical Appendix Figure 1). A total of 451 serum samples were collected and subjected to IgG ELISA by using an *Escherichia coli*–derived recombinant nucleocapsid (N) protein of NiV (NiV-N-ELISA) (*2*). Two Leschenault’s rousette bats (*Rousettus leschenaulti*) were vaccinated with the same recombinant N protein to obtain positive serum specimens that contained antibodies against NiV-N. An optical density of 492 nm for negative control serum (1,000× dilution) was designated as 1:1,000 ELISA units. ELISA titers of sample serum specimens were obtained at a single dilution (1,000×) by using a standard curve of positive serum with high titers. A sample titer >3,000 was considered positive for IgG against NiV.

Positive results were detected from only 2 fruit bat species, *R. leschenaulti* (31 bats [49.1%]) and *Cynopterus sphinx* (3 bats [2.8%]) (Table). Of the 34 samples positive by ELISA, only 22 (20 from the former and 2 from the latter bat species), which had enough volume left, were further analyzed by Western blot (WB) with *E. coli*–expressed recombinant N protein of NiV (Table; Technical Appendix Figure 2). Twenty of the 22 specimens were confirmed as positive by WB. ELISA-positive samples with high titers were also positive by WB for both bat species. However, only 1 sample from an *R. leschenaulti* bat was positive by WB that used a baculovirus-expressed recombinant N protein. Because of the different protein expression systems, the reactivity of bat antibody against NiV protein in WBs showed different patterns (Technical Appendix Figure 2). Neutralization tests (NTs) in which live NiVs (strain Ma-JMR-01–98) were used were performed at the Institute of Tropical Medicine, Nagasaki University. No specimens of *C. sphinx* bats were positive by NT; however, 2 specimens from *R. leschenaulti* bats, both positive with low titers, were confirmed to be positive by NT (50% cytopathic effect after NT, titers of 33.6 and 14.1). However, the latter specimen was negative by WB analysis.

**Table:** Results of serologic tests for Nipah virus on bats captured in Vietnam, 2007–2008*

Bat species	No. samples	No. (%) ELISA+	No. WB+/no. ELISA+	No. micro-NT+/no. ELISA+†
Megachiroptera				
* Cynopterus sphinx*	109	3 (2.8)	2/2	0/3
* Rousettus leschenaulti*	74	31 (41.9)	18/20	2‡/31
Total	183	34 (18.6)	20/22	2/34
Microchiroptera				
* Chaerephon plicata*	130	0		
* Hipposideros armiger*	1	0		
* Hipposideros cineraceus*	3	0		
* Hipposideros larvatus*	3	0		
* Hipposideros pomona*	5	0		
* Hypsugo cadornae*	25	0		
* Megaderma spasma*	3	0		
* Miniopterus magnater*	1	0		
* Scotophilus kuhi*	45	0		
Unidentified	52	0		
Total	268	0		

*+, positive; WB, Western blot; NT, neutralization test. Blank cells indicate that test was not done. †Micro-NT was done only on specimens positive for Nipah virus by ELISA. ‡One of the NT-positive samples was negative by WB analysis.

Seroepidemiologic studies in other countries have indicated that *Pteropus* spp. bats (fruit-eating bats) are the main reservoirs for NiV (*3**–**6*). Pteropid bats are usually found only in southern Vietnam. We could not obtain these bats for our study. However, a relatively high prevalence (49.1%) of henipavirus antibody was found in *R. leschenaulti* specimens from Hoa Binh Province. *Rousettus* spp. bats are the only megabats that use echolocation. These bats hang together on cave ceilings in a tightly packed manner and in groups composed of bats of both sexes and of different ages. They roost in large colonies and fly vast distances to find fruit (*7*). This behavior might be related to their high rate of seropositivity for viral infections. In southern China, bats of the same species showed a high prevalence of henipavirus antibody (*8*). *R. leschenaulti* bats are distributed from central to northern Vietnam and southern China. *C. sphinx* bats are common all over Vietnam, and their habitat overlaps with that of pteropid bats in southern Vietnam.

Previous studies showed that IgG ELISA results for NiV-positive flying foxes correlated well with NT results (*3**,**4*). However, in our study, discrepancies existed between NT results and NiV-N-ELISA and WB results. A reason for these differences could be that Nipah-like viruses are circulating among bats in Vietnam, producing antibodies that are cross-reactive by ELISA and WB, but poorly cross-reactive by NT. The cross-reactive antibodies were probably not directed against neutralizing epitopes. To date, no reports have been made of an increased number of febrile encephalitis cases among the residents in Hoa Binh and Dak Lak Provinces where seropositive bats were captured. The circulating viruses may lack the pathogenic potential of Hendra and Nipah viruses.

A survey by questionnaire was conducted among residents of Dak Nong and Dak Lak Provinces, where NiV-N-ELISA–positive *C. sphinx* bats were captured, to determine the frequency of contact between humans and bats. Risk factors for infection were observed in this study, such as bat hunting and cooking and drinking bat blood. In such situations, persons have direct contact with bat body fluids and feces and might be bitten during bat hunting. Thus, long-term systematic surveillance of bats is needed to determine the ecologic relationship between bats, humans, other animals, and the environment.

## Supplementary Material

Technical Appendix Bat study sites in Vietnam, 2007–2008.
