# Supplementary material for: Serologic Evidence of Nipah Virus Infection in Bats, Vietnam
Source: Emerg Infect Dis. 2012 Mar;18(3):536–7. doi: 10.3201/eid1803.111121 (PMC3309638; doi:10.3201/eid1803.111121)
Supplement: Technical Appendix — Bat study sites in Vietnam, 2007–2008. [file 11-1121-Techapp_2p.pdf]

Article DOI: http://dx.doi.org/10.3201/eid1803.111121

Serologic Evidence of Nipah Virus Infection in Bats, Vietnam

Technical Appendix

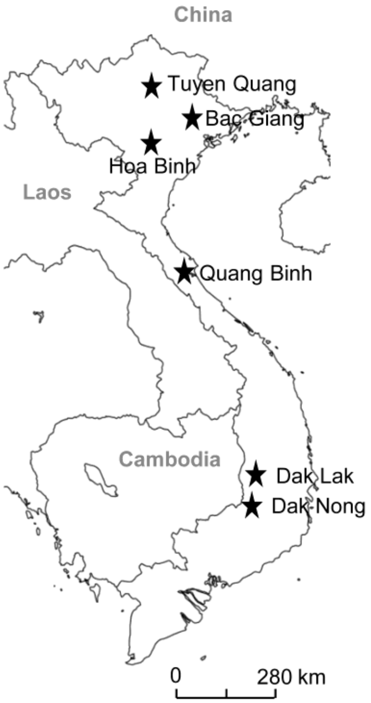

Technical Appendix Figure 1. Bat study sites in Vietnam, 2007–2008.

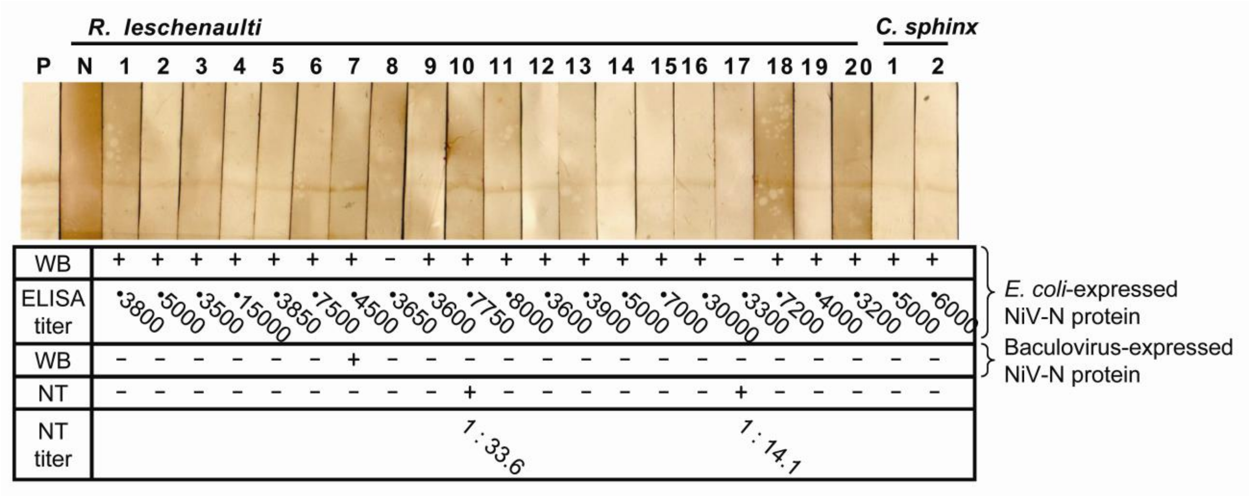

Technical Appendix Figure 2. Bat specimen (from *Rousettus leschenaulti* and *Cynopterus sphinx* bats) results of WB analysis and ELISA that used *Escherichia coli*–expressed and baculovirus-expressed recombinant NiV N protein and virus NT, Vietnam, 2007–2008. WB, Western blot; NiV, Nipah virus; N, nucleocapsid; NT, neutralization test; P, positive control.
